# Two‐Dimensional Conjugated Metal Organic Frameworks with Dual Metal Catalytic Active Sites Enhance POD Activity for Antibacterial Treatment

**DOI:** 10.1002/advs.76605

**Published:** 2026-08-05

**Authors:** Yixuan Lu, Qinqin Li, Yumin Chen, Shihan Zhang, Shuai Ding, Yanpei Wang, Youxing Liu, Yaru Guo, Xuliang Deng, Shaojun Guo

**Affiliations:** ^1^ Department of Geriatric Dentistry, School and Hospital of Stomatology, School of Materials Science and Engineering Peking University Beijing P. R. China; ^2^ Beijing Key Laboratory of Electrochemical Process and Technology for Materials Beijing University of Chemical Technology Beijing P. R. China; ^3^ Peking University Hospital of Stomatology Sanya Division (Sanya Stomatology Center) Sanya Hainan P. R. China

**Keywords:** 2D cMOFs, antibacterial, oral ulcers, POD activity

## Abstract

The emergence of drug‐resistant bacteria has brought a grand challenge to the treatment of oral ulcers (OUs). Peroxidase (POD) enzymes catalyze the conversion of hydrogen peroxide (H_2_O_2_) into reactive oxygen species (ROS), offering a promising strategy for treating drug‐resistant bacterial infections and oral wound therapy. Among them, two‐dimensional conjugated metal‐organic frameworks (2D‐cMOFs) with high π‐π conjugated structure and efficient electron transport capability serve as potent POD mimics, enhancing ROS generation for targeted antimicrobial applications. However, the reported single‐metal‐center 2D cMOFs possess the intrinsically weak adsorption interaction with H_2_O_2_, usually decreasing the conversion of H_2_O_2_ to ROS, exhibiting relatively low enzymatic activity and antibacterial performance. Herein, we reported a new class of Ni doped Cu 2D cMOFs, defined as CuNi‐HHTP 2D‐cMOFs, for constructing dual activity site to enhance the adsorption and activation of H_2_O_2_, which significantly facilitates the treatment of OUs. Mechanism investigation reveals the introduction of Ni atom upshifts the d‐band center of CuNi‐HHTP 2D‐cMOFs from ‐2.563 to ‐2.084 eV, which enhances the adsorption and activation of H_2_O_2_, endowing its 5.0‐fold enhancement in bactericidal efficiency. Besides, the as‐made CuNi‐HHTP 2D‐cMOFs show improved antibacterial performance in vivo, which significantly promote OU healing, with macroscopic wound closure on the 7^th^ day.

## Graphical abstract

A new class of Cu, Ni bimetallic 2D cMOF (CuNi‐HHTP 2D cMOF) featuring dual catalytic sites and high delocalized electronic structure for boosting POD performance. The CuNi‐HHTP 2D‐cMOF enhances the conversion kinetics of H_2_O_2_ to ROS, which subsequently enhances antibacterial activity and accelerates the healing of oral ulcers, with macroscopic wound closure on the 7th day.

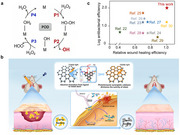

## 1 Introduction

**FIGURE 1:**
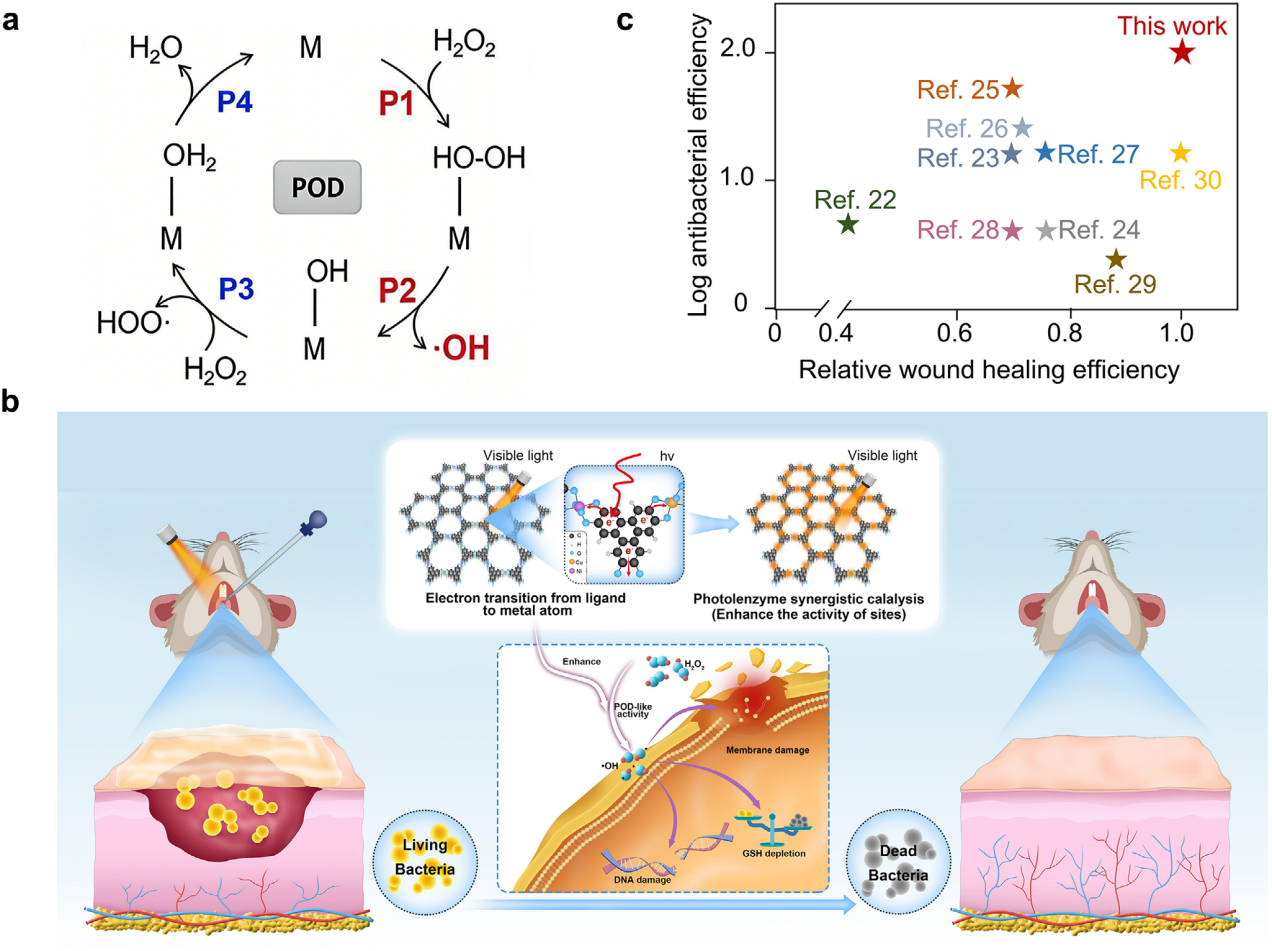
Design, working mechanisms of CuNi‐HHTP 2D‐cMOFs in this study. (a) Design of CuNi‐HHTP 2D‐cMOFs. (b) Antibacterial mechanism. (c) Disinfection performance comparison of CuNi‐HHTP 2D‐cMOFs with reported photocatalysts.

Oral ulcers (OUs) affect over 20% of the population and diminish patients’ quality of life [1, 2]. These lesions are frequently painful, interfere with speaking and eating, restrict nutrition, and substantially impair oral and systemic health [3]. The human oral cavity contains complex microbiota, changes in which are affected by many factors such as exogenous food intake, physical diseases, and drug treatment [4]. It has been speculated that dynamic changes in oral microbiota is an even more important initiator of OUs [5, 6]. Therefore, antibiotics are extensively employed in clinical settings for OU treatment, unfortunately leading to the emergence of drug‐resistant bacteria [7, 8, 9]. In contrast, reactive oxygen species (ROS)‐mediated bactericidal mechanisms do not induce resistance development, with hydroxyl radicals (·OH) being the most potent oxidizing agents [10, 11, 12]. Biological peroxidase (POD) enzymes, which catalyze the generation of ·OH from hydrogen peroxide (H_2_O_2_), present a promising strategy for combating drug‐resistant bacteria and OU treatment [13, 14]. However, biological enzymes possess certain limitations, including limited reusability, high cost, and stability issues [15]. In comparison, nanozymes offer advantages such as reusability, cost‐effectiveness, stability, and precise control over morphology and composition [16, 17]. The main steps of generating ·OH in POD reaction include substrate absorption and substrate decomposition, corresponding to P1 and P2 in Figure 1a [13, 16]. Conventional three‐dimensional non‐conjugated frameworks suffer from sluggish photogenerated charge‐carrier migration and severe charge recombination, thereby limiting their catalytic and antibacterial efficiency. Two‐dimensional conjugated metal‐organic frameworks (2D‐cMOFs) integrate atomic‐thin morphology, rapid in‐plane charge transport, and uniformly dispersed metal nodes, which fully exposes coordinately unsaturated metal sites and achieves near‐theoretical atomic utilization efficiency [18, 19]. This structural advantage synergistically enhances H_2_O_2_ decomposition and ·OH generation, delivering exceptional POD performance across both P1 and P2 systems. However, the reported single metal center 2D‐cMOFs possess the weak adsorption interaction with H_2_O_2_, decreasing the conversion of H_2_O_2_ to ROS, which exhibit relatively low enzymatic activity and antibacterial performance [20, 21].

To address the aforementioned challenges, herein, we report a new class of Cu, Ni bimetallic cMOF (CuNi‐HHTP 2D‐cMOF) for facilitating spatial separation of photogenerated carriers and accordingly boosting POD performance. We demonstrate that the incorporation of Ni upshifts the d‐band center of CuNi‐HHTP 2D‐cMOFs, thereby facilitating H_2_O_2_ adsorption in P1 and promoting the photogenerated electron transfer for H_2_O_2_ decomposition and ·OH generation in P2 (Figure 1a). Consequently, the CuNi‐HHTP MOFs exhibit a 6.0‐fold increase in ROS generation and a 5.0‐fold enhancement in bactericidal efficiency of methicillin‐resistant *Staphylococcus aureus* (*MRSA*) compared to Cu‐HHTP MOFs. Furthermore, owing to its outstanding in vivo antibacterial activity and capacity to mitigate infection‐triggered inflammation, CuNi‐HHTP 2D‐cMOFs effectively accelerate oral wound healing, with macroscopic wound closure achieved by day 7 (Figure 1b). Notably, this synergistic integration of robust bactericidal efficacy and superior wound‐healing performance represents a marked advancement over previously reported comparable systems (Figure 1c) [22, 23, 24, 25, 26, 27, 28, 29, 30].

## 2 Results

### 2.1 Synthesis and Characterization of CuNi‐HHTP 2D‐cMOFs

**FIGURE 2:**
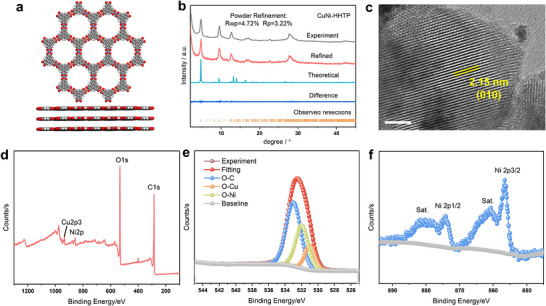
The characterization of CuNi‐HHTP 2D‐cMOFs. (a) Simulated crystal structural model of CuNi‐HHTP 2D‐cMOFs. (b) PXRD pattern and pawley refinement results of CuNi‐HHTP MOFs. (c) High‐resolution TEM image of CuNi‐HHTP 2D‐cMOFs, scale bar: 10 nm. (d) XPS survey spectra of CuNi‐HHTP 2D‐cMOFs, (e) High‐resolution O 1s and (f) Ni 2p XPS spectra of CuNi‐HHTP 2D‐cMOFs.

The crystal structures of Cu‐HHTP and CuNi‐HHTP 2D‐cMOFs were investigated using powder X‐ray diffraction (PXRD), transmission electron microscopy (TEM), and X‐ray photoelectron spectroscopy (XPS). As illustrated in the simulated molecular structures (Figure 2a and Figure S1), the asymmetric unit of Cu‐HHTP comprises three metal atoms and two HHTP ligands, whereas the metal atoms are coordinated with four oxygen atoms of HHTP organic ligands. Both Cu‐HHTP and CuNi‐HHTP 2D‐cMOFs possess a highly conjugated planar structure with unit cell parameters of a = b = 21.75 Å, c = 6.71 Å, α = β = 60°, and γ = 120°. The experimental PXRD patterns of CuNi‐HHTP MOFs and Cu‐HHTP MOFs (Figure 2b and Figure S2) reveal that the diffraction peaks located at 4.75°, 9.45°, 12.56°, and 28.31° can be indexed to the (100), (200), (210), and (002) crystal planes, respectively. These patterns match well with the simulated ones for the AA stacking model, with Rwp = 4.72% and Rp = 3.22% for CuNi‐HHTP, and Rwp = 4.16% and Rp = 3.55% for Cu‐HHTP (Tables S1 and S2). TEM images of CuNi‐HHTP 2D‐cMOFs display the lattice spacing of 2.1 nm (Figure 2c and Figure S3), corresponding to the (100) crystal plane, which is consistent with both the simulated molecular structure.

XPS results show the presence of C, O, Cu element in the Cu‐HHTP 2D‐cMOFs, and the C, O, Cu, Ni elements in the CuNi‐HHTP 2D‐cMOFs (Figure 2d and Figure S4). The high‐resolution C 1s spectrum (Figure S5) shows a prominent peak at 285.4 eV and 284.8 eV, assigned to C─O bonds and C ═ C bonds, respectively. The high‐resolution C 1s spectrum (Figure S5) shows a prominent peak at 285.4 eV, assigned to C─O bonds, which confirm the presence of HHTP organic molecular evidences the successful coordination of the HHTP ligand with metal centers. The high‐resolution (HR) O 1s spectrum of Cu‐HHTP and CuNi‐HHTP 2D‐cMOFs (Figure 2e and Figure S6) shows a prominent peak at 530.7 eV, assigned to O─Cu bonds, and the peak at 531.2 eV of CuNi‐HHTP 2D‐cMOFs originates from O─Ni bond, which evidences the successful coordination of the HHTP ligand with metal centers. The HR Cu 2p spectra of Cu‐HHTP and CuNi‐HHTP 2D‐cMOFs have two peaks at 933.5 eV and 944.2 eV, which are attributed to Cu 2p_3/2_ and Cu 2p_1/2_, respectively. In the HR Ni 2p XPS spectra of CuNi‐HHTP, the binding energies at 859.2 eV and 879.9 eV correspond to 2p_3/2_ and Ni 2p_1/2_, respectively (Figure 2f and Figure S7). Notably, the Cu 2p_3/2_ peak of Cu‐HHTP (933.5 eV) shows a positive shift relative to that of metallic Cu (932.2 eV), while the Ni 2p_3/2_ peak of CuNi‐HHTP (859.2 eV) is also positively shifted relative to metallic Ni (852.6 eV), further indicating strong metal–oxygen coordination.

### 2.2 Photocatalytic POD‐Like Activity of CuNi‐HHTP MOFs

**FIGURE 3:**
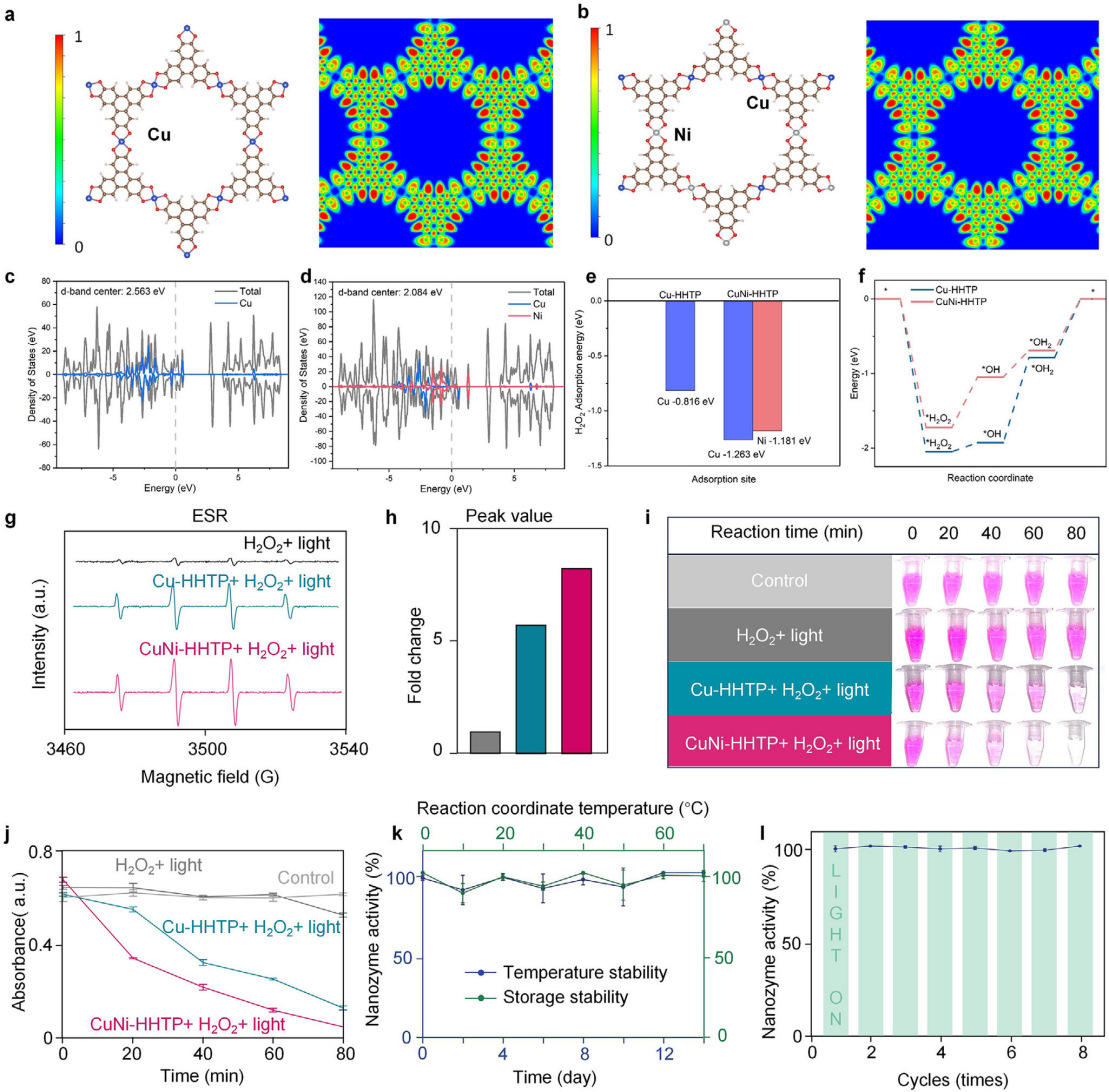
Theoretical calculations and photocatalytic properties of CuNi‐HHTP MOFs. The ELF diagrams of Cu‐HHTP 2D‐cMOFs (a) and CuNi‐HHTP 2D‐cMOFs (b). TDOS and PDOS of Cu‐HHTP 2D‐cMOFs (c) and CuNi‐HHTP 2D‐cMOFs (d). (e) The adsorption energy of H_2_O_2_ on Cu‐HHTP MOFs and CuNi‐HHTP MOFs. (f) Energy change diagram of photocatalytic H_2_O_2_ decomposition on Cu‐HHTP MOFs and CuNi‐HHTP MOFs. (g) ESR spectra of pure H_2_O_2_+light system, Cu‐HHTP+H_2_O_2_+light system, CuNi‐HHTP+H_2_O_2_+light system. (h) Quantitative analysis of ESR spectra in (g). (i, j) Images and degradation curve of RhB and in pure H_2_O_2_+light system, Cu‐HHTP+H_2_O_2_+light system, CuNi‐HHTP+H_2_O_2_+light system. (k) The nanozyme activity in a long term storageand in different temperatures. (l) Multiple operational cycles activity of CuNi‐HHTP MOFs nanozyme.

To reveal the effect of Ni incorporation on the electronic structure, the electron localization function (ELF) was used to reveal the pronounced electron delocalization around both Cu and Ni sites, which is beneficial for the adsorption of H_2_O_2_, identifying them as the primary active centers for H_2_O_2_ adsorption (Figure 3a and b). The projected density of states for CuNi‐HHTP MOFs (PDOS, Figure 3c and d) shows the emergence of a new peak at 1.30 eV, indicating the formation of a Ni‐O bond. In addition, the d‐band center of CuNi‐HHTP 2D‐cMOFs shifted upward from ‐2.563 eV to ‐2.084 eV relative to Cu‐HHTP 2D‐cMOFs, suggesting stronger adsorbate‐substrate interactions between H_2_O_2_ and CuNi‐HHTP 2D‐cMOFs, confirming that the introduction of Ni enhances the adsorption of H_2_O_2_ over CuNi‐HHTP MOFs, which is beneficial for the conversion of H_2_O_2_ to ROS. Moreover, the calculated adsorption energies of H_2_O_2_ on Cu and Ni sites in CuNi‐HHTP 2D‐cMOFs are ‐1.263 eV and ‐1.181 eV respectively, which are significantly higher than that of Cu site in Cu‐HHTP (‐0.816 eV), confirming that the introduction of Ni atom enhances the adsorption of H_2_O_2_. Furthermore, the energy change diagram of photocatalytic H_2_O_2_ decomposition shows that the reaction free energy of photocatalytic H_2_O_2_ to produce ROS over CuNi‐HHTP 2D‐cMOFs is significantly lower than that of Cu‐HHTP MOFs, indicating that the introduction of Ni decreases the reaction free energy of the conversion of H_2_O_2_ to ROS (Figure 3f), thus enhancing antibacterial activity of CuNi‐HHTP 2D‐cMOFs.

Further, H_2_O_2_ fluorescence probe was used to detect the consumption of the H_2_O_2_ in the reaction system (Figure S8). The result shows that the consumption of H_2_O_2_ with CuNi‐HHTP is 3 times quicker than with Cu‐HHTP in the first 20 minutes. The electron spin resonance (ESR) was used to study the ·OH concentration in pure H_2_O_2_+light system, Cu‐HHTP+H_2_O_2_+light system, and CuNi‐HHTP+H_2_O_2_+light system. Results show that the ·OH signals in CuNi‐HHTP+H_2_O_2_ +light system and Cu‐HHTP+H_2_O_2_+light system are 8.3‐ and 5.7‐fold higher than that of pure H_2_O_2_+light system (Figure 3g, h), which confirm the superior catalytic efficiency of CuNi‐HHTP 2D‐cMOFs. The rhodamine B (RhB) was added into the reaction system to assess the ·OH formation kinetics based on the fact that the ·OH with strong oxidizing capacity can degrade RhB. In the CuNi‐HHTP+H_2_O_2_+light system, the color of the solution changes red to completely colorless within 80 min, while the others show a gradual shift from red to pink (Figure 3i). The RhB degradation rate in the CuNi‐HHTP group was significantly higher than that of Cu‐HHTP group, suggesting that the introduction of Ni enhance the conversion of H_2_O_2_ to ·OH radical. Notably, the CuNi‐HHTP 2D‐cMOFs exhibit significantly higher reaction kinetics within the initial 20‐minute phase (Figure 3j), demonstrating a first‐order rate constant (k) of 0.036 min^−1^, sixfold greater than that of Cu‐HHTP (0.006 min^−1^) under identical conditions. Furthermore, the as‐synthesized CuNi‐HHTP 2D‐cMOFs demonstrate robust nanozyme activity across a temperature range of 0 C–70°C (Figure 3k) and maintain exceptional catalytic stability over repeated reaction cycles (Figure 3l).

### 2.3 The Antibacterial Capacity of CuNi‐HHTP Against Methicillin‐Resistant *Staphylococcus Aureus* and *Escherichia coli* In Vitro

**FIGURE 4:**
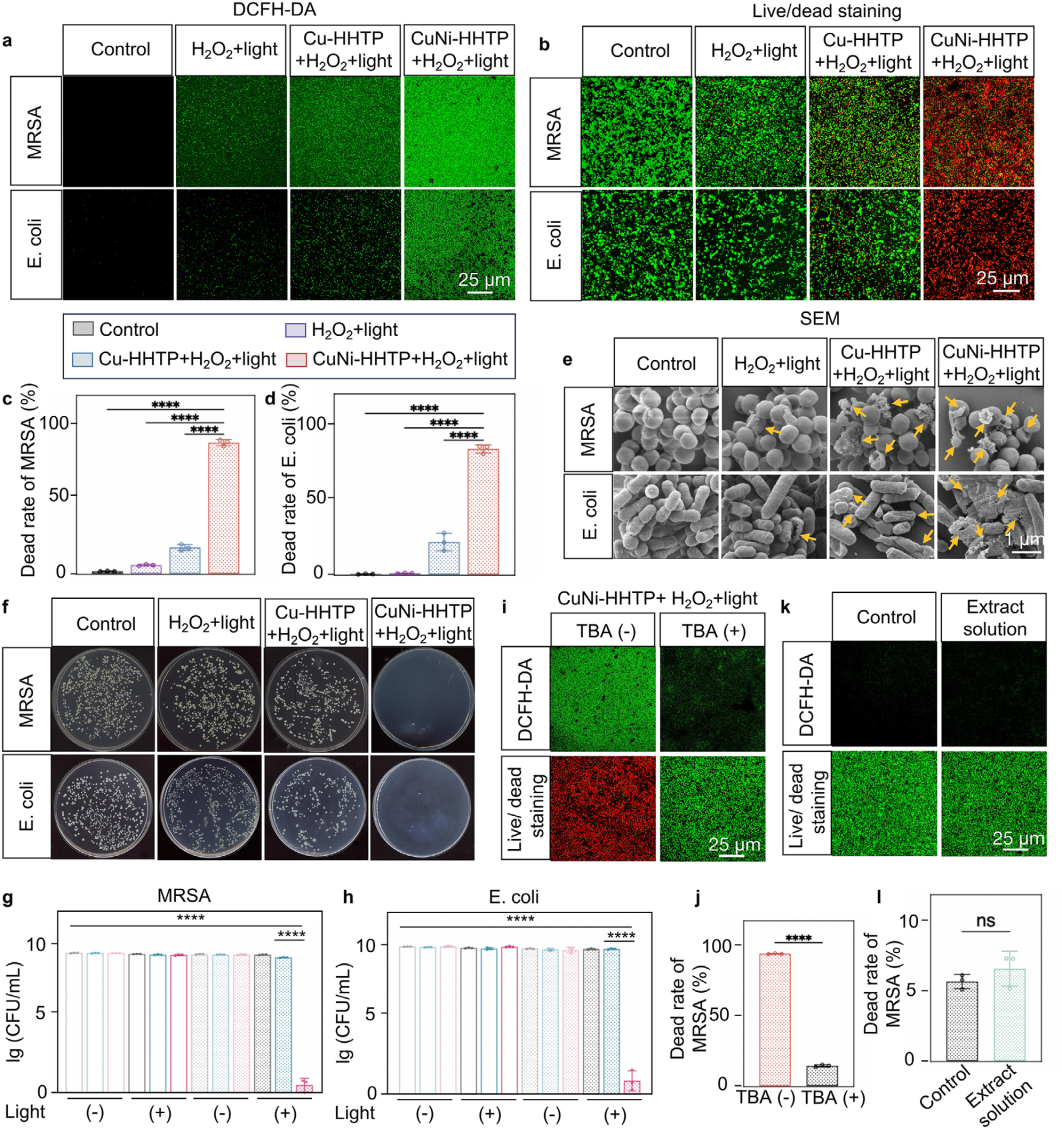
Antibacterial activity evaluation of CuNi‐HHTP MOFs in vitro. (a) Fluorescence images of ROS in *MRSA* and *E. coli*. (b) Live/dead staining images of *MRSA* and *E. coli*. (c) Dead rate quantification of *MRSA*. (d) Dead rate quantification of *E. coli*. (e) SEM images of *MRSA* and *E. coli*. (f) Photos of agar plates of bacterial colonies *MRSA* and *E. coli*. Logarithm of colony forming unit (CFU) counting of *MRSA* (g) and *E. coli* (h) after grouped treatment. (i) Fluorescence images of ROS in *MRSA* and live/dead staining images of *MRSA* treated with TBA. (j) Dead rate of *MRSA* treated with TBA. (k) Fluorescence images of ROS in *MRSA* and live/dead staining images of *MRSA* treated with extract solution. (l) Dead rate of *MRSA* treated with extract solution. Data are presented as mean ± SD. Statistical significance was determined by one‐way ANOVA with Tukey's post hoc test. ns indicate p > 0.05, ****p < 0.0001.

The antibacterial activity of nanozymes was assessed using methicillin‐resistant *Staphylococcus aureus* (*MRSA*) and *Escherichia coli* (*E. coli*), common pathogen in clinical [31]. We demonstrated that the H_2_O_2_ and visible light radiation are activation factors for efficient antibacterial. Notably, ROS concentration in *MRSA* and *E. coli* in CuNi‐HHTP+H_2_O_2_ +light group are 37.4‐ and 4.8‐fold enhancement compared to the control groups, respectively (Figure 4a, Figures S9–S12). Moreover, the ROS level in *MRSA* and *E. coli* in CuNi‐HHTP+H_2_O_2_ +light group are 2.1‐ and 2.4‐fold to Cu‐HHTP+H_2_O_2_+light group, thus endowing CuNi‐HHTP 2D‐cMOFs with augmented catalytic activity. Consequently, the antibacterial efficiency of CuNi‐HHTP 2D‐cMOFs is approximately 4 times improvement to both bacteria compared to Cu‐HHTP 2D‐cMOFs (Figure 4b–d, Figures S13–S16). Consistent with the fact that ROS can directly disrupt cell membranes and kill bacteria, SEM images reveal severe membrane damage of *MRSA* and *E. coli* in CuNi‐HHTP+H_2_O_2_+light group (Figure 4e, Figure S17), further confirming the ROS‐based antibacterial mechanism. The colony formation capability of bacteria was also detected. At an equivalent dilution factor (10^6^), the control group yielded hundreds of colonies after 24 hours, whereas the CuNi‐HHTP+H_2_O_2_+light resulted in a complete absence of detectable *MRSA* and *E. coli* colonies, indicating near‐complete bacterial eradication (Figure 4f–h, Figure S18). By contrast, the residual bacterial load of Cu‐HHTP+H_2_O_2_+light group remained within the range of 10^8^.

Furthermore, to verify the effect of ·OH generated by CuNi‐HHTP in antibacterial activity, a hydroxyl radical scavenging group was added. After adding hydroxyl radical scavenger–tert‐butanol (TBA, 50mM) with CuNi‐HHTP+H_2_O_2_+light, the intracellular ROS level was decreased in bacteria, and the live/dead staining results exhibited live rate of 85.4% for *MRSA* (Figure 4i,j, Figure S19). To explore the possible contribution of released metal ions, bacterial suspensions were co‐cultured with the corresponding extract solution of CuNi‐HHTP 2D‐cMOFs (Figure 4k, l, Figure S20). The extract solution showed no significant effects in bacterial dead rate to the control group. It indicates that the antibacterial effect is not mainly caused by released metal ions. Hence, the enhanced antibacterial activity should mainly be attributed to the ·OH generation induced by the nanozymes under light catalyze. However, dynamically monitoring the ROS‐generating and antibacterial activities of CuNi‐HHTP during bacterial growth and treatment requires further exploration alongside with the pH changes within infectious microenvironment.

In addition, live/dead cell staining (Figure S21) and cell counting kit‐8 (CCK‐8, Figure S22) assays demonstrated the biocompatibility of CuNi‐HHTP 2D‐cMOFs and CuNi‐HHTP+H_2_O_2_+light system, exhibiting no significant effects on the survival and proliferation of human buccal mucosa fibroblasts (hBMFs) in 7 days. The biocompatibility of ions released by CuNi‐HHTP 2D‐cMOFs was also tested (Figures S23, S24). The examination of intracellular ROS level and expression of fibroblast‐associated genes of hBMFs were also performed (Figures S25, S26).

### 2.4 Healing Efficacy of CuNi‐HHTP 2D‐cMOFs on Infected OUs in the Rat Models

**FIGURE 5:**
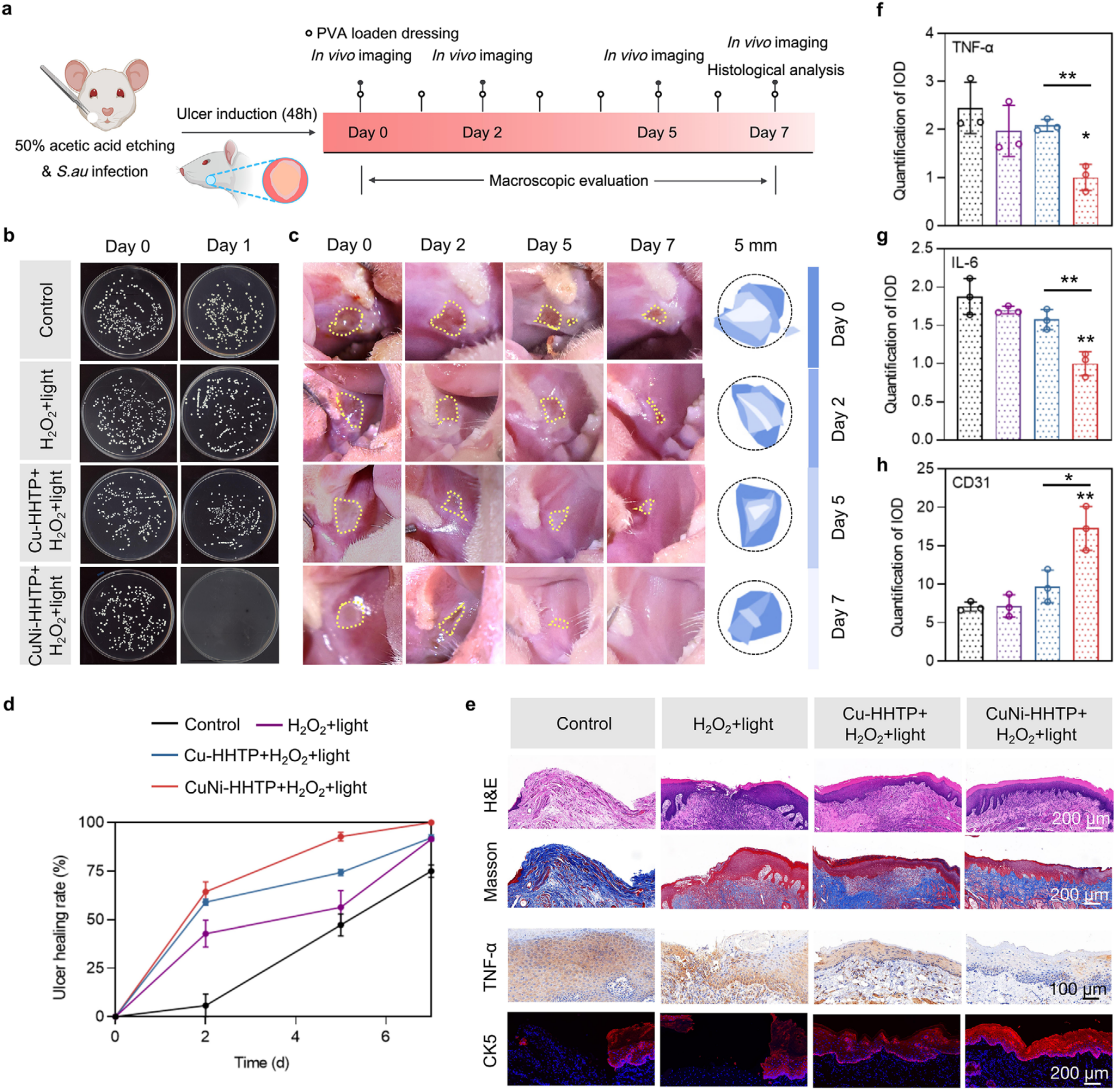
Antibacterial activity evaluation of CuNi‐HHTP 2D‐cMOFs in vivo. (a) Schematic procedure of infected OU model and treatment. (b) *MRSA* colonies pictures from wound surface. (c) Images of the addressed OUs and simulation of ulcer morphological transformations. (d) Comparative analysis of the relative ulcer closure rate among different groups. (e) H&E staining, Masson staining, IHC staining of TNF‐α and IF staining of CK5 of the infected OUs after grouped treatment. (f–h) Quantitative analysis of integral optical density (IOD) of TNF‐α (f), IL‐6 (g), and CD31 (h). Data are presented as mean ± SD. Statistical significance was determined by one‐way ANOVA with Tukey's post hoc test. * p < 0.05, **p < 0.01.

After determining the improved catalytic efficiency and in vitro antibacterial activity of CuNi‐HHTP 2D‐cMOFs, the therapeutic application in infectious OUs was further evaluated. To this end, 8‐week‐old male Sprague‐Dawley rats were employed to establish an oral mucosal ulcer model. Ulcers measuring 5 mm^2^ were induced using 50% acetic acid and subsequently infected with *MRSA*. The resulting infected lesions were then treated with either polyvinyl alcohol (PVA, 300 mg mL^−1^) alone or a mixture of CuNi‐HHTP nanozymes (500 µg mL^−1^) and PVA (Figure 5a). Moreover, a daily treatment with 3% H_2_O_2_ and visible light was applied to all groups except the control group. PVA was used as a biocompatible carrier matrix to retain the therapeutic components at the wound site and facilitate local treatment. It was not designed as an active antibacterial component in this study. The blank control group was used to evaluate spontaneous wound healing, while the H_2_O_2_+light group served as a procedural control to assess the influence of H_2_O_2_ and light irradiation in the absence of CuNi‐HHTP. To confirm the biosafety of CuNi‐HHTP 2D‐cMOFs, H&E staining for major organs (heart, liver, spleen, lung and kidney) and serum biochemistry indicators of liver functions and renal functions were also carried out (Figures S27, S28).

The coating dilution plate experiments showed that the number of bacteria abruptly reduced in CuNi‐HHTP group after 24 h treatment, which is superior to other groups (Figure 5b). Representative images of the infected wounds in different groups (Figure 5c) demonstrated that new tissue formation was significantly increased by the CuNi‐HHTP 2D‐cMOFs. As shown in Figure 5d, the CuNi‐HHTP+H_2_O_2_+light group achieved an ulcer closure rate of 92.8% on day 5, markedly higher than the control (47.3%), H_2_O_2_+light(56.3%), and Cu‐HHTP+H_2_O_2_+light (74.3%) groups. Notably, the macroscopic wound closure of OUs was observed in the CuNi‐HHTP group by Day 7.

H&E, Masson staining and immunohistochemical staining were performed for the histological assessment of newly formed mucosa tissue. As shown in Figure 5e, both the CuNi‐HHTP and Cu‐HHTP groups displayed nearly epithelial regeneration along with well‐organized collagen fibers (blue indicates type I collagen in Masson staining), closely resembling healthy buccal mucosa. In contrast, the control and H_2_O_2_ groups exhibited only partial tissue repair. Notably, compared with the Cu‐HHTP group, the CuNi‐HHTP treatment resulted in a more uniform epithelial thickness and longer, more regularly arranged epithelial rete ridges.

Immunohistochemical staining of key pro‐inflammatory cytokines, including interleukin‐6 (IL‐6) and tumor necrosis factor‐α (TNF‐α), revealed a pronounced suppression of inflammatory factor expression in the CuNi‐HHTP+H_2_O_2_+light group (Figure 5e and Figure S29), whereas TNF‐α and IL‐6 levels remained elevated in the control group. Relative quantitative results displayed that the integral optical density (IOD) of TNF‐α and IL‐6 of CuNi‐HHTP+H_2_O_2_+light group is 0.4‐, 0.5‐fold to control group (Figure 5f and g). Furthermore, cytokeratin‐5 (CK5) immunofluorescence staining demonstrated epithelial coverage in CuNi‐HHTP+H_2_O_2_+light group, with CK5 appropriately distributed within the basal and intermediate layers (Figure 5e). For further analysis, the relative quantitative of IOD of CK5 is 3.4 times of the control group (Figure S30). Besides, the expression level of CD31 associated with angiogenesis in the newly form tissue almost 4.3‐, 2.4‐ fold to the control, H_2_O_2_+light and Cu‐HHTP+H_2_O_2_+light group (Figure 5h and Figure S29). The decreased IL‐6 expression, increased CD31‐positive staining, and enhanced CK5 expression suggested that the CuNi‐HHTP+H_2_O_2_+light treatment alleviated inflammation, promoted angiogenesis, and facilitated re‐epithelialization during healing process. These histological and immunofluorescence results should be interpreted as downstream therapeutic outcomes of the combined treatment rather than direct evidence distinguishing the independent contribution of each component. Collectively, the CuNi‐HHTP+H_2_O_2_+light group showed the most favorable infectious mucosal healing outcome, suggesting that the therapeutic effect was mainly associated with the material‐mediated photocatalytic POD‐like activity.

Although the present study demonstrated the superior therapeutic efficacy of CuNi‐HHTP+H_2_O_2_+light in vivo, the split contribution of each component was not fully investigated in the animal model. In particular, additional in vivo groups, including PVA alone, CuNi‐HHTP alone, H_2_O_2_ alone, visible light alone, CuNi‐HHTP+H_2_O_2_, and CuNi‐HHTP+light, would further clarify the individual and synergistic effects of each component. This represents a limitation of the current study, and more detailed component‐resolved in vivo experiments will be performed in future work. Furthermore, H&E staining and immunofluorescence analysis of IL‐6, CD31, and CK5 suggested reduced inflammation, enhanced angiogenesis, and promoted re‐epithelialization, but long‐term tissue remodeling, epithelial barrier recovery, collagen organization, recurrence, and functional restoration were not evaluated. Therefore, the present study mainly demonstrates accelerated early‐stage antibacterial repair of infected oral ulcers, and more comprehensive long‐term studies are needed to assess complete tissue regeneration.

## 3 Conclusions

In summary, to overcome the limited therapeutic efficacy of conventional MOFs against oral ulcers, caused by their inherently weak photocatalytic POD‐like activity, we developed a new class of 2D CuNi‐HHTP cMOFs featuring dual active sites that promote the conversion of H_2_O_2_ into ROS, thereby markedly enhancing the therapeutic efficacy against OUs. We demonstrate that the introduction of Ni atom upshifts the d‐band center of CuNi‐HHTP 2D‐cMOFs from ‐2.563 to ‐2.084 eV, which enhances the adsorption of H_2_O_2_ and promotes the conversion of H_2_O_2_ to ROS. The rate constant for H_2_O_2_ conversion to ROS over CuNi‐HHTP 2D‐cMOFs is 0.036 min^−1^, as determined by the first‐order rate constant of RhB degradation, which is 6.0‐folds higher than that of Cu‐HHTP 2D‐cMOFs. The high conversion kinetics of H_2_O_2_ to ROS endow CuNi‐HHTP 2D‐cMOFs with antibacterial efficiency of 6.9*10^5^ CFU min^−1^, representing a 5.0‐fold improvement over Cu‐HHTP cMOFs. Therefore, CuNi‐HHTP 2D‐cMOFs exhibits an accelerated wound repair of OUs, achieving macroscopic wound closure by day 7. Overall, this work reports a new strategy of Ni regulated electronic structure of Cu‐HHTP cMOFs for enhancing the conversion of H_2_O_2_ to ROS for promoting the treatment efficacy of OUs, which has guiding significance for rational design of efficient catalysts.

## 4 Methods

### 4.1 Chemical Materials

2,3,6,7,10,11‐Triphenylenehexol (HHTP), N‐methyl‐2‐pyrrolidone (NMP), CuSO_4_·5H_2_O and NiSO_4_·6H_2_O were purchased from Shanghai Macklin Biochemical Co., Ltd. and Energy Chemical Co. Ltd. without further purification.

### 4.2 Synthesis of Cu‐HHTP and CuNi‐HHTP

Cu‐HHTP was synthesized according to a reported procedure [32]. Briefly, 21.62 mg of HHTP ligand, 24.96 mg of CuSO_4_·5H_2_O were dissolved in 5 mL of deionized water. The mixture was sonicated for 30 minutes to ensure complete dissolution. This was followed by a reaction at 80°C for 48 hours in a glass vial after the addition of 0.3 mL of NMP, which yielded dark yellow crystals. The final product was collected by centrifugation, washed, and dried.

CuNi‐HHTP was prepared according to the same synthetic procedure as Cu‐HHTP, except that 24.96 mg CuSO_4_·5H_2_O were replaced by 12.48 mg CuSO_4_·5H_2_O, and 13.14 mg of NiSO_4_·6H_2_O.

### 4.3 Characterization

The molecular structure and unit cell parameters of Cu‐HHTP and CuNi‐HHTP were obtained by the Material Studio software. Powder X‐ray diffraction (PXRD) test was performed using a Bruker D2 Phaser operating at 40 kV voltage and 15 mA current with Cu‐Kα X‐ray radiation (λ = 0.154056 nm). Transmission electron microscopy (TEM, Tecnai G2 F20 S‐Twin, FEI) was utilized to obtain the morphology of the samples. X‐ray photoelectron spectroscopy (XPS) analysis was measured on the Shimadzu Kratos AXIS UltraDLD system using the contaminant carbon (C1s = 284.8 eV) as a reference.

### 4.4 Theoretical Calculation Details

All Density Functional Theory (DFT) calculations were performed using the Vienna Ab Initio Simulation Package (VASP). The exchange functional was applied utilizing the generalized gradient approximation (GGA) of Perdew‐Burke‐Ernzerhof (PBE) functional, and use the plane‐wave basis with a kinetic energy cutoff of 500 eV to account for valence electrons. To better simulate the actual surface situation, the vacuum spacing in a direction perpendicular to the plane of the catalyst was 20 Å to separate the surface slab from its periodic duplicates. The self‐consistent calculations applied a convergence energy threshold of 10^−5^ eV. The equilibrium lattice constants were optimized with maximum stress on each atom within 0.02 eV/Å.

### 4.5 Reactive Oxygen Species Detection

Reactive oxygen species (ROS) generated by the materials were assessed using Rhodamine B degradation and electron spin resonance (ESR) techniques. To evaluate the ROS production, samples were mixed with 1 mL of Rhodamine B solution (8 µg mL^−1^) containing H_2_O_2_ (50 µM). The absorbance of the resulting supernatants was recorded using a UV–vis spectrophotometer (Molecular Devices, USA) over a wavelength range of 400–700 nm. ROS were identified using an ESR spectrometer (Bruker, Germany). To capture the ·OH radicals formed during the reaction between materials and H_2_O_2_, 5,5‐dimethyl‐1‐pyrroline‐N‐oxide (DMPO, 0.1 mM, DOJINDO, Japan) was used as a spin‐trapping agent. H_2_O_2_ levels was measured using a H_2_O_2_ specific fluorescence probe, Peroxy Orange 1 (5µM; HY‐103469, MCE). The MOFs (50µg mL^−1^) were mixed into 1% H_2_O_2_ under light irradiation. The fluorescence was detected by the fluorescence microplate reader (excitation light  =  543 nm, emission light  =  565 nm).

### 4.6 Inductively Coupled Plasma Mass Spectrometer (ICP‐MS)

Concentrations of Cu and Ni were quantified using an Agilent 7800 ICP‐MS instrument. The instrument operating parameters were set as: RF power 1550 W, nebulizer gas flow 1.00 L/min, auxiliary gas flow 1.00 L/min, peristaltic pump rate 20 r/min, and sample flush time 40 s. All samples were pre‐acid digested prior to measurement; external calibration was applied for quantitative calculation, with procedural blanks included to correct background interference. Solutions were produced by PBS mixed with MOFs (50 µg mL^−1^) in 37°C after being centigrade.

### 4.7 Bacterial Culture

*MRSA* and *E. coli* was cultured in Luria‐Bertani (LB) medium (pH 7.0) under 37°C incubation. The bacterial suspension was then standardized to approximately 10^8^ CFU mL^−1^ prior to experimental use.

### 4.8 Antibacterial Properties In Vitro

LB broth was prepared with or without Cu‐HHTPs and CuNi‐HHTPs (50 µg mL^−1^). Intracellular reactive oxygen species (ROS) generation in bacteria was quantified using the fluorescent probe DCFH_2_‐DA (10 µM, MCE, China). After co‐incubation of bacteria with different media and H_2_O_2_ for 1 h, cells were collected, washed with sterile PBS (pH 7.4), and incubated with DCFH‐DA at 37°C in the dark. The oxidized fluorescent product DCF was detected using excitation/emission wavelengths around 488/525–535 nm. Fluorescence imaging was conducted using a confocal laser scanning microscope (CLSM; Leica, Germany).

To assess antibacterial performance, bacteria suspensions were exposed to materials‐containing media supplemented with H_2_O_2_ for 1 h. The treated bacteria were then stained with a Live/Dead Bacterial Staining Kit (Yeasen, China) and visualized by CLSM. Antibacterial efficacy was further quantified through colony‐counting plate assays.

For morphological characterization, bacterial samples were fixed with 4% glutaraldehyde (Solarbio, China) for 1 h, followed by dehydration through a graded ethanol series. The microstructural features were subsequently examined using a scanning electron microscope (SEM; JEOL, Japan).

Serial dilutions of bacterial suspension were prepared with sterile phosphate‐buffered saline (PBS). An aliquot of 10 µL diluted bacterial solution was evenly spread onto solid agar plates using a sterile spreader. All plates were incubated at 37°C overnight under aerobic conditions. Colony‐forming units (CFU) were counted manually, and the viable bacterial density was calculated based on dilution folds. Three parallel replicates were set for each group.

Tert‐butanol (TBA, MCE, 50 mM) was used to scavenge ·OH. Extract solutions were produced by medium mixed with MOFs (50 µg mL^−1^) after being centigrade and sterile.

### 4.9 Cell Culture

Human buccal mucosa fibroblasts (hBMFs) were cultured in DMEM (Procell, China) at 37°C under 5% CO_2_ in a humidified incubator. Once the cells reached a confluency of 80–90%, they were detached using 0.25% trypsin‐EDTA (Procell, China) for 60 seconds and centrifuged at 1000 rpm for 5 minutes. The resulting cell pellets were re‐suspended in fresh medium and evenly distributed. Cells at passages 4 were plated onto well plates.

### 4.10 Cytotoxicity Assays

Cell culture medium was supplemented with or without Cu‐HHTPs and CuNi‐HHTPs (50 µg mL^−1^). Cell viability was evaluated using the Live/Dead Viability/Cytotoxicity Kit (Beyotime, China). After 24 h of incubation, cells were stained according to the manufacturer's protocol and imaged using confocal laser scanning microscopy (CLSM).

The proliferation of HBMFs was assessed using the Cell Counting Kit‐8 (CCK‐8, DOJINDO, Japan). Cells were seeded in plates, and after 8 h of attachment, the medium was replaced with CCK‐8 working solution. Following 2 h of incubation, supernatants were collected and their absorbance at 450 nm was measured using a microplate reader (Molecular Devices, USA). Cells were then rinsed twice with PBS and cultured in fresh DMEM. These procedures were repeated at predetermined time points to monitor cell growth dynamics. Extract solutions were produced by medium mixed with MOFs (50 µg mL^−1^) after being centigrade and sterile.

### 4.11 Quantitative Real‐Time Polymerase Chain Reaction (RT‐qPCR)

Total RNA was isolated from cells using a commercial RNA extraction kit. After removing genomic DNA contamination, purified RNA was reverse transcribed into complementary DNA (cDNA) using a reverse transcription kit. RT‐qPCR was performed with SYBR Green qPCR Master Mix on a real‐time PCR system. Primer sequences were listed in Table S3.

### 4.12 In Vivo Infectious Oral Ulcers Model

All animal surgical procedures were approved by the Institutional Animal Care and Use Committee of Peking University (DLASBE0691).

Male Sprague‐Dawley rats (8 weeks old) were anesthetized via intraperitoneal injection tribromoethanol. Oral ulcers were established by applying 50% acetic acid–soaked filter paper (5 × 5 mm) to the buccal mucosa for 3 min, which caused immediate mucosal blanching. 10 µL of *MRSA* suspension (10^8^ CFU mL^−1^) was then applied onto the wound of each rat. Two days after ulcer induction (defined as day 0), the rats were randomly divided into four groups: a polyvinyl alcohol (PVA, 300 mg mL^−1^) control group and three experimental groups. The experimental groups received treatments of PVA alone, PVA containing Cu‐HHTPs (500 µg mL^−1^), or PVA containing CuNi‐HHTPs (500 µg mL^−1^). The experimental groups were further treated daily with 3% H_2_O_2_ and exposed to xenon light irradiation for 10 minutes. The dressings were applied every day. For the first two days, bacterial counts were measured using the colony‐counting plate method. The bacteria were collected from oral ulcers surface by cotton swab and then dipped it into 1 mL PBS. The plates were coated with 10 µL bacteria solution without further dilution. On days 0, 2, 5, and 7, ulcer healing was recorded photographically before each application. Wound closure rates were quantified using ImageJ software. On days 7, histological assessments were conducted, including H&E staining, Masson staining, and immunohistochemical staining for IL‐6 and TNF‐α. Additionally, immunofluorescence staining of CK5 and CD31 was performed. The integral optical density (IOD) of the immunohistochemical images was quantified using Image‐Pro Plus 6.0 software. Blood samples were collected from the orbital sinus at days 7 post‐treatment, centrifuged at 3000 × g for 15 min at 4°C to obtain serum. Serum levels of aspartate transaminase (AST), total bilirubin (T‐Bil), blood urea nitrogen (BUN) and creatinine (CREA) were measured using an automatic biochemical analyzer (Hitachi 7600, Japan) according to the manufacturers’ instructions.

### 4.13 Statistical Analysis

All experiments were independently repeated three times as biological replicates. Quantitative data were presented as mean ± SD (n = 3 independent biological replicates), with error bars representing variability. Statistical analyses were performed using Student's t‐test or one‐way ANOVA in GraphPad Prism (version 10.0). Statistical significance was indicated as follows: *p < 0.05, **p < 0.01, ***p < 0.001, ****p < 0.0001.

## Author contributions

**S. Guo**, **Y. Guo**, **X. Deng** and **Y. Liu** conceived the project. **Y. Lu**, **Q. Li** and **Y. Chen** contributed equally to this work. **Y. Lu** and **Q. Li** designed and performed the experiments. **Y. Chen** conducted data analyses with the help of **S. Zhang**, **S. Ding**, **Y. Wang**, **Y. Liu**, **S. Guo**, **Y. Guo** and **X. Deng**. All the authors participated in discussions of the research.

## Conflicts of Interest

The authors declare no conflicts of interest.

## Supporting information




**Supporting File**: advs76605‐sup‐0001‐SuppMat.docx.

## Data Availability

The data that support the findings of this study are available from the corresponding author upon reasonable request.
