# Supplementary material for: Two‐Dimensional Conjugated Metal Organic Frameworks with Dual Metal Catalytic Active Sites Enhance POD Activity for Antibacterial Treatment
Source: Adv Sci (Weinh). 2026 Aug 5;13(56):e76605. doi: 10.1002/advs.76605 (PMC13440200; doi:10.1002/advs.76605)
Supplement: Supplementary file 1 [file ADVS-13-e76605-s001.docx]

Supporting Information

**Two-Dimensional Conjugated Metal Organic Frameworks with Dual Metal Catalytic Active Sites Enhance POD activity for Antibacterial Treatment**

*Yixuan Lu#^1^, Qinqin Li#^2^, Yumin Chen#^1^, Shihan Zhang^1^, Shuai Ding^1^, Yanpei Wang^1^, Youxing Liu*^2^, Yaru Guo*^1,3^, Xuliang Deng*^1^, Shaojun Guo*^1^*

Figure S1-S30

Table S1-S3

**Fig. S1** The crystal structural model of Cu-HHTP MOFs.

**Fig. S2** PXRD pattern and Pawley refinement of Cu-HHTP MOFs.

**Fig. S3** High-resolution TEM image of Cu-HHTP MOFs, scale bar: 10 nm.

**Fig. S4** The XPS survey spectra of Cu-HHTP MOFs.

**Fig. S5** High-resolution C 1s spectra of Cu-HHTP MOFs.

**Fig. S6** High-resolution O 1s spectra of Cu-HHTP MOFs.

**Fig. S7** High-resolution Cu 2p XPS spectra of (a) Cu-HHTP and (b) CuNi-HHTP MOFs.

**Fig. S8** The consumption of H_2_O_2_ over reaction time.

**Fig. S9** Fluorescence images of ROS in *MRSA*.

**Fig. S10** Relative quantification of ROS in *MRSA*.

**Fig. S11** Fluorescence images of ROS in *E. coli*.

**Fig. S12** Relative quantification of ROS in *E. coli*.

**Fig. S13** Live/dead staining images of *MRSA*.

**Fig. S14** Dead rate quantification of *MRSA*.

**Fig. S15** Live/dead staining images of *E. coli.*

**Fig. S16** Dead rate quantification of *E. coli*.

**Fig. S17** SEM images of MRSA and *E. coli*.

**Fig. S18** Agar plates of bacterial colony formation of *MRSA* and *E. coli*.

**Fig. S19** Relative fluorescence quantification of ROS in *MRSA* treated by ·OH scavenger.

**Fig. S20** Relative fluorescence quantification of ROS in *MRSA* treated by extract solution of CuNi-HHTP.

**Fig. S21** Live/dead staining of hBMFs treated with nanozymes.

**Fig. S22** Cell viability of hBMFs treated with nanozymes in 7 days.

**Fig. S23** Ion release curve of CuNi-HHTP in 7 days.

**Fig. S24** Cell viability of hBMFs treated by extract solution of CuNi-HHTP in 7 days.

**Fig. S25** Intracellular ROS levels measured by DCFH-DA in fibroblast. (a) Representative fluorescent images. (b) Quantitative fluorescence intensity of DCFH-DA.

**Fig. S26** The relative mRNA levels of genes related to fibroblast functions, including COL1 (a), COL3 (b), FN1 (c), and FGF2 (d).

**Fig. S27** H&E staining for major organs (heart, liver, spleen, lung and kidney) in each group.

**Fig. S28** Serum biochemistry indicators of liver functions and renal functions, including ALT, AST, BUN, T-Bil and CREA.

**Fig. S29** IHC staining of IL-6 and IF staining of CD31 of the infected oral ulcers after grouped treatment.

**Fig. S30** Quantitative analysis of integral optical density (IOD) of CK5.

**Tab. S1** The atomic fractional coordinates for Cu-HHTP.

| a = b = 21.75 Å, c = 6.71 Å, α = β = 60°, γ = 120°, | | | |
| --- | --- | --- | --- |
| Atom | X | Y | Z |
| C1 | 0.49126 | 0.82368 | 0.01701 |
| C2 | 0.55613 | 0.88802 | 0.01697 |
| C3 | 0.62034 | 0.88802 | 0.0169 |
| C4 | 0.62087 | 0.82368 | 0.01691 |
| C5 | 0.55611 | 0.75792 | 0.01696 |
| C6 | 0.55611 | 0.69258 | 0.01692 |
| C7 | 0.62087 | 0.69158 | 0.01691 |
| C8 | 0.62034 | 0.62671 | 0.01685 |
| C9 | 0.55613 | 0.5625 | 0.01681 |
| C10 | 0.49127 | 0.56197 | 0.01684 |
| C11 | 0.49026 | 0.62673 | 0.01691 |
| C12 | 0.42492 | 0.62673 | 0.01694 |
| C13 | 0.35916 | 0.56197 | 0.01691 |
| C14 | 0.29482 | 0.56249 | 0.01693 |
| C15 | 0.29482 | 0.6267 | 0.01699 |
| C16 | 0.35915 | 0.69157 | 0.01702 |
| C17 | 0.42492 | 0.69258 | 0.01699 |
| C18 | 0.49025 | 0.75792 | 0.01699 |
| C19 | 0.01938 | 0.48898 | 0.01678 |
| C20 | 0.01937 | 0.42477 | 0.01672 |
| C21 | 0.95504 | 0.35991 | 0.01665 |
| C22 | 0.88927 | 0.3589 | 0.01664 |
| C23 | 0.88927 | 0.42476 | 0.01668 |
| C24 | 0.95504 | 0.48951 | 0.01676 |
| C25 | 0.82394 | 0.42476 | 0.01666 |
| C26 | 0.82394 | 0.29357 | 0.01662 |
| C27 | 0.75808 | 0.3589 | 0.01665 |
| C28 | 0.69333 | 0.35991 | 0.01664 |
| C29 | 0.69385 | 0.42478 | 0.01665 |
| C30 | 0.75807 | 0.48899 | 0.01666 |
| C31 | 0.82293 | 0.48952 | 0.01667 |
| C32 | 0.82293 | 0.2278 | 0.01661 |
| C33 | 0.75806 | 0.16346 | 0.01665 |
| C34 | 0.69385 | 0.16346 | 0.0167 |
| C35 | 0.69332 | 0.2278 | 0.01669 |
| C36 | 0.75808 | 0.29357 | 0.01665 |
| O1 | 0.08481 | 0.55195 | 0.0169 |
| O2 | 0.0848 | 0.42723 | 0.01676 |
| O3 | 0.22939 | 0.49952 | 0.01687 |
| O4 | 0.22939 | 0.62424 | 0.01701 |
| O5 | 0.63088 | 0.09803 | 0.01678 |
| O6 | 0.55858 | 0.95345 | 0.017 |
| O7 | 0.68331 | 0.95345 | 0.01682 |
| O8 | 0.7556 | 0.09803 | 0.01665 |
| O9 | 0.55859 | 0.49953 | 0.01676 |
| O10 | 0.63088 | 0.42724 | 0.01664 |
| O11 | 0.75561 | 0.55196 | 0.01667 |
| O12 | 0.68332 | 0.62425 | 0.01684 |
| Cu1 | 0.1571 | 0.52573 | 0.01689 |
| Cu2 | 0.65709 | 0.02574 | 0.01682 |
| Cu3 | 0.6571 | 0.52574 | 0.01673 |
| H1 | 0.43864 | 0.82411 | 0.01706 |
| H2 | 0.67393 | 0.82412 | 0.01688 |
| H3 | 0.67412 | 0.744 | 0.01694 |
| H4 | 0.43864 | 0.50892 | 0.01682 |
| H5 | 0.35835 | 0.50872 | 0.01687 |
| H6 | 0.35872 | 0.74419 | 0.01706 |
| H7 | 0.95547 | 0.30728 | 0.01661 |
| H8 | 0.95586 | 0.54276 | 0.01679 |
| H9 | 0.64008 | 0.30748 | 0.01663 |
| H10 | 0.87556 | 0.54257 | 0.01668 |
| H11 | 0.87555 | 0.22737 | 0.01657 |
| H12 | 0.64027 | 0.22737 | 0.01672 |

**Tab. S2** The atomic fractional coordinates for CuNi-HHTP.

| a = 37.36 Å, b = 21.57 Å, c = 6.71 Å, α = β = γ = 90° | | | |
| --- | --- | --- | --- |
| Atom | X | Y | Z |
| C1 | 0.98299 | 0.42195 | 0.75437 |
| C2 | 0.98303 | 0.39005 | 0.72193 |
| C3 | 0.9831 | 0.42215 | 0.68983 |
| C4 | 0.98309 | 0.48676 | 0.68956 |
| C5 | 0.98304 | 0.52014 | 0.72194 |
| C6 | 0.98308 | 0.58547 | 0.72194 |
| C7 | 0.98309 | 0.61886 | 0.68956 |
| C8 | 0.98315 | 0.68346 | 0.68983 |
| C9 | 0.98319 | 0.71557 | 0.72193 |
| C10 | 0.98316 | 0.68367 | 0.75436 |
| C11 | 0.98309 | 0.6184 | 0.75487 |
| C12 | 0.98306 | 0.58573 | 0.78754 |
| C13 | 0.98309 | 0.61761 | 0.82042 |
| C14 | 0.98307 | 0.58492 | 0.85259 |
| C15 | 0.98301 | 0.52071 | 0.85259 |
| C16 | 0.98298 | 0.48801 | 0.82042 |
| C17 | 0.98301 | 0.51988 | 0.78754 |
| C18 | 0.98301 | 0.48721 | 0.75487 |
| C19 | 0.98322 | 0.52071 | 0.99031 |
| C20 | 0.98328 | 0.58492 | 0.99032 |
| C21 | 0.98335 | 0.11761 | 0.52248 |
| C22 | 0.98336 | 0.08574 | 0.55536 |
| C23 | 0.98332 | 0.01988 | 0.55536 |
| C24 | 0.98324 | 0.98801 | 0.52248 |
| C25 | 0.98334 | 0.98721 | 0.58803 |
| C26 | 0.98338 | 0.1184 | 0.58803 |
| C27 | 0.98335 | 0.02014 | 0.62096 |
| C28 | 0.98336 | 0.98676 | 0.65333 |
| C29 | 0.98335 | 0.92215 | 0.65307 |
| C30 | 0.98334 | 0.89005 | 0.62096 |
| C31 | 0.98333 | 0.92194 | 0.58853 |
| C32 | 0.98339 | 0.18367 | 0.58853 |
| C33 | 0.98335 | 0.21557 | 0.62097 |
| C34 | 0.9833 | 0.18347 | 0.65307 |
| C35 | 0.98331 | 0.11886 | 0.65334 |
| C36 | 0.98335 | 0.08547 | 0.62096 |
| C37 | 0.98299 | 0.92195 | 0.25437 |
| C38 | 0.98303 | 0.89005 | 0.22193 |
| C39 | 0.9831 | 0.92215 | 0.18983 |
| C40 | 0.98309 | 0.98675 | 0.18956 |
| C41 | 0.98304 | 0.02014 | 0.22194 |
| C42 | 0.98308 | 0.08548 | 0.22194 |
| C43 | 0.98309 | 0.11886 | 0.18956 |
| C44 | 0.98315 | 0.18346 | 0.18983 |
| C45 | 0.98319 | 0.21557 | 0.22193 |
| C46 | 0.98316 | 0.18366 | 0.25436 |
| C47 | 0.98309 | 0.1184 | 0.25487 |
| C48 | 0.98306 | 0.08573 | 0.28754 |
| C49 | 0.98309 | 0.11761 | 0.32042 |
| C50 | 0.98307 | 0.08492 | 0.35259 |
| C51 | 0.98301 | 0.02071 | 0.35259 |
| C52 | 0.98298 | 0.98801 | 0.32042 |
| C53 | 0.98301 | 0.01988 | 0.28754 |
| C54 | 0.98301 | 0.98721 | 0.25487 |
| C55 | 0.98322 | 0.02071 | 0.49031 |
| C56 | 0.98328 | 0.08492 | 0.49031 |
| C57 | 0.98335 | 0.61761 | 0.02248 |
| C58 | 0.98336 | 0.58573 | 0.05536 |
| C59 | 0.98332 | 0.51988 | 0.05536 |
| C60 | 0.98324 | 0.48801 | 0.02248 |
| C61 | 0.98334 | 0.48721 | 0.08803 |
| C62 | 0.98338 | 0.6184 | 0.08803 |
| C63 | 0.98335 | 0.52014 | 0.12096 |
| C64 | 0.98336 | 0.48676 | 0.15333 |
| C65 | 0.98335 | 0.42215 | 0.15307 |
| C66 | 0.98334 | 0.39005 | 0.12096 |
| C67 | 0.98333 | 0.42195 | 0.08854 |
| C68 | 0.98339 | 0.68366 | 0.08853 |
| C69 | 0.98335 | 0.71557 | 0.12097 |
| C70 | 0.9833 | 0.68347 | 0.15307 |
| C71 | 0.98331 | 0.61886 | 0.15334 |
| C72 | 0.98335 | 0.58547 | 0.12096 |
| O1 | 0.9831 | 0.49046 | 0.9576 |
| O2 | 0.98324 | 0.61517 | 0.9576 |
| O3 | 0.98313 | 0.61518 | 0.8853 |
| O4 | 0.98299 | 0.49046 | 0.8853 |
| O5 | 0.98322 | 0.21741 | 0.68456 |
| O6 | 0.983 | 0.32584 | 0.72071 |
| O7 | 0.98318 | 0.38821 | 0.65835 |
| O8 | 0.98335 | 0.27977 | 0.6222 |
| O9 | 0.98324 | 0.77977 | 0.7207 |
| O10 | 0.98336 | 0.8882 | 0.68456 |
| O11 | 0.98333 | 0.82585 | 0.62219 |
| O12 | 0.98316 | 0.71741 | 0.65834 |
| O13 | 0.9831 | 0.99046 | 0.45759 |
| O14 | 0.98324 | 0.11517 | 0.4576 |
| O15 | 0.98313 | 0.11518 | 0.38531 |
| O16 | 0.98299 | 0.99046 | 0.38531 |
| O17 | 0.98322 | 0.71741 | 0.18456 |
| O18 | 0.983 | 0.82584 | 0.22071 |
| O19 | 0.98318 | 0.88821 | 0.15834 |
| O20 | 0.98335 | 0.77977 | 0.1222 |
| O21 | 0.98324 | 0.27976 | 0.2207 |
| O22 | 0.98336 | 0.3882 | 0.18456 |
| O23 | 0.98333 | 0.32585 | 0.12219 |
| O24 | 0.98316 | 0.21741 | 0.15834 |
| Ni1 | 0.98311 | 0.55282 | 0.92145 |
| Ni2 | 0.98327 | 0.80281 | 0.67145 |
| Ni3 | 0.98311 | 0.05282 | 0.42145 |
| Cu1 | 0.98318 | 0.30281 | 0.67145 |
| Cu2 | 0.98318 | 0.80281 | 0.17145 |
| Cu3 | 0.98327 | 0.30281 | 0.17145 |
| H1 | 0.98294 | 0.39521 | 0.78068 |
| H2 | 0.98312 | 0.51285 | 0.66303 |
| H3 | 0.98306 | 0.59306 | 0.66294 |
| H4 | 0.98318 | 0.7104 | 0.78068 |
| H5 | 0.98313 | 0.67046 | 0.82082 |
| H6 | 0.98294 | 0.43517 | 0.82064 |
| H7 | 0.98339 | 0.17046 | 0.52226 |
| H8 | 0.98321 | 0.93517 | 0.52207 |
| H9 | 0.98337 | 0.01256 | 0.67996 |
| H10 | 0.98332 | 0.89521 | 0.56222 |
| H11 | 0.98343 | 0.21041 | 0.56222 |
| H12 | 0.98328 | 0.09276 | 0.67986 |
| H13 | 0.98294 | 0.89521 | 0.28068 |
| H14 | 0.98312 | 0.01284 | 0.16304 |
| H15 | 0.98306 | 0.09306 | 0.16294 |
| H16 | 0.98318 | 0.2104 | 0.28068 |
| H17 | 0.98313 | 0.17046 | 0.32082 |
| H18 | 0.98294 | 0.93517 | 0.32064 |
| H19 | 0.98339 | 0.67046 | 0.02226 |
| H20 | 0.98321 | 0.43517 | 0.02207 |
| H21 | 0.98337 | 0.51256 | 0.17996 |
| H22 | 0.98332 | 0.39521 | 0.06222 |
| H23 | 0.98343 | 0.7104 | 0.06222 |
| H24 | 0.98328 | 0.59277 | 0.17986 |
|  |  |  |  |

**Tab. S3** Primers sequence of qPCR.

| Gene name | Primer | Sequence |
| --- | --- | --- |
| Homo GAPDH | Forward | CTTTGGTATCGTGGAAGGACTC |
| Homo GAPDH | Reverse | GTAGAGGCAGGGATGATGTTCT |
| Homo COL1 | Forward | GGCAAAGATGGACTCAACGG |
| Homo COL1 | Reverse | ATCATCAGCCCGGTAGTAGC |
| Homo COL3 | Forward | GAGAACCTGGTGCAAATGGG |
| Homo COL3 | Reverse | TTCCCATCACTTCCTGGTCC |
| Homo FGF2 | Forward | AGGAGTGTGTGCTAACCGTT |
| Homo FGF2 | Reverse | CAGTTCGTTTCAGTGCCACA |
| Homo FN1 | Forward | CCTACCTAGGCAATGCGTTG |
| Homo FN1 | Reverse | TTCATGGCAGCGGTTTGCGA |
